# *Pcdh19* Loss-of-Function Increases Neuronal Migration *In Vitro* but is Dispensable for Brain Development in Mice

**DOI:** 10.1038/srep26765

**Published:** 2016-05-31

**Authors:** Daniel T. Pederick, Claire C. Homan, Emily J. Jaehne, Sandra G. Piltz, Bryan P. Haines, Bernhard T. Baune, Lachlan A. Jolly, James N. Hughes, Jozef Gecz, Paul Q. Thomas

**Affiliations:** 1 School of Biological Sciences, The University of Adelaide, Adelaide, South Australia 5005, Australia; 2 Robinson Research Institute, University of Adelaide, Adelaide, South Australia 5005, Australia; 3 School of Medicine, The University of Adelaide, Adelaide, South Australia 5005, Australia

## Abstract

Protocadherin 19 (*Pcdh19*) is an X-linked gene belonging to the protocadherin superfamily, whose members are predominantly expressed in the central nervous system and have been implicated in cell-cell adhesion, axon guidance and dendrite self-avoidance. Heterozygous loss-of-function mutations in humans result in the childhood epilepsy disorder *PCDH19* Girls Clustering Epilepsy (*PCDH19* GCE) indicating that *PCDH19* is required for brain development. However, understanding *PCDH19* function *in vivo* has proven challenging and has not been studied in mammalian models. Here, we validate a murine *Pcdh19* null allele in which a *β-Geo* reporter cassette is expressed under the control of the endogenous promoter. Analysis of *β-Geo* reporter activity revealed widespread but restricted expression of *PCDH19* in embryonic, postnatal and adult brains. No gross morphological defects were identified in *Pcdh19*^+/*β-Geo*^ and *Pcdh19*^Y/*β-Geo*^ brains and the location of *Pcdh19* null cells was normal. However, *in vitro* migration assays revealed that the motility of *Pcdh19* null neurons was significantly elevated, potentially contributing to pathogenesis in patients with *PCDH19* mutations. Overall our initial characterization of *Pcdh19*^+*/β-Geo*^, *Pcdh19*^*β-Geo/β-Geo*^ and *Pcdh19*^*Y/β-Geo*^mice reveals that despite widespread expression of *Pcdh19* in the CNS, and its role in human epilepsy, its function in mice is not essential for brain development.

Protocadherins comprise a large family of single pass transmembrane glycoproteins that have important roles in cell-cell adhesion, dendrite self-avoidance and axon guidance. In mammals, ~70 protocadherin genes have been identified, the majority of which are located in three genomic “clusters” termed α, β and γ. The remainder, termed “non-clustered protocadherins” are scattered throughout the genome and are classified as δ1, δ2 and “other”. Non-clustered protocadherins are widely expressed in the central nervous system and have been implicated in homotypic cell adhesion, neuronal migration and synaptic plasticity^1^,^2^,^3^,^4^.

*Pcdh19* is an X-chromosome linked member of the δ2 protocadherin subfamily and is highly expressed in the developing mouse cortex and hippocampus^5^. *PCDH19* is highly conserved across human, mouse, zebrafish and chicken and has weak homotypic cell adhesion properties. It has been shown to interact with N-cadherin and members of the WAVE complex (Nap1 and Cyfip2) suggesting a role in actin cytoskeletal dynamics^6^,^7^. Genetic disruption of the closely related *Pcdh10* or *Pcdh17* genes (members of the δ2 subfamily) in mice have revealed roles in axon extension, regulation of presynaptic assembly and growth of striatal axons and thalamocortical projections^8^,^9^,^10^. These studies raise the possibility *Pcdh19* functions similarly during mouse brain development; however this remains unexplored due to the lack of a validated *Pcdh19* null mouse.

The majority of studies investigating the endogenous role of *Pcdh19* have been performed in zebrafish^11^,^12^,^13^. These studies have utilized either morpholino knockdown or mutation of *Pcdh19* with each approach producing different phenotypes. Morpholino knockdown results in a severe phenotype where cell motility in the neural plate is compromised resulting in a severe disruption to early brain morphogenesis^13^. In contrast, *pcdh19* null zebrafish generated by TALEN-induced frame-shift mutation are viable and fertile, and exhibit disruption of columnar architecture in the optic tectum resulting in impaired visually guided behaviours^12^. While these data support a role for *pcdh19* in cell-cell recognition during zebrafish brain development, the discordance between the mutant and morphant phenotypes, which is commonly observed in zebrafish^14^, makes interpretation of these data difficult.

In humans, heterozygous *PCDH19* mutations cause *PCDH19* GCE^5^,^15^ which is now recognized as the second most common cause of monogenic epilepsy^16^. Mutations of *PCDH19* that cause *PCDH19* GCE are mainly missense, however as some cases have complete gene deletions, all pathogenic mutations are almost certainly loss-of-function. The phenotype of *PCDH19* GCE is variable – affected girls present with symptoms ranging from benign focal epilepsy with normal cognitive function to severe seizures and intellectual disability that resemble Dravet syndrome^16^,^17^. The inheritance pattern of *PCDH19* GCE is highly unusual for an X-linked gene, and is described as X-linked dominant with male sparing i.e. heterozygous females are affected whereas hemizygous males are not. Due to random X-chromosome inactivation affected females are composed of a mosaic population of normal and *PCDH19* mutant cells. Intriguingly, rare cases of affected males have also been described, which arise from somatic mutation and also display mosaicism^5^,^18^. It has been proposed that the mosaicism of normal *PCDH19* and mutant expressing cells leads to ‘scrambling’ of the neuronal circuitry in the brain of affected individuals. However, experimental support for this ‘cellular interference’ model remains limited.

Here we investigate the *in vivo* expression and function of *Pcdh19* using mutant mice where the *Pcdh19* gene is disrupted by a *β-galactosidase-neomycin* (*β-Geo*) reporter cassette. At this allele, *β-Geo* was expressed under the control of the endogenous *Pcdh19* promoter, whilst *Pcdh19* expression itself was ablated (i.e. a knock-out allele). Using *β-Geo* expression and X-Gal staining we show that *Pcdh19* expression is widely expressed in the developing CNS through to adulthood. Although brain morphology in *Pcdh19*^*Y/β-Geo*^*, Pcdh19*^*β-Geo/β-Geo*^ and *Pcdh19*^+*/β-Geo*^ mutants appears grossly normal, *in vitro* analysis indicates that *Pcdh19* null neurons have a slight but significant increase in motility. These data suggest more subtle roles for *Pcdh19* beyond establishment of gross brain architecture.

## Results

### Generation and validation of a *Pcdh19* null mouse model

**Figure 1:**
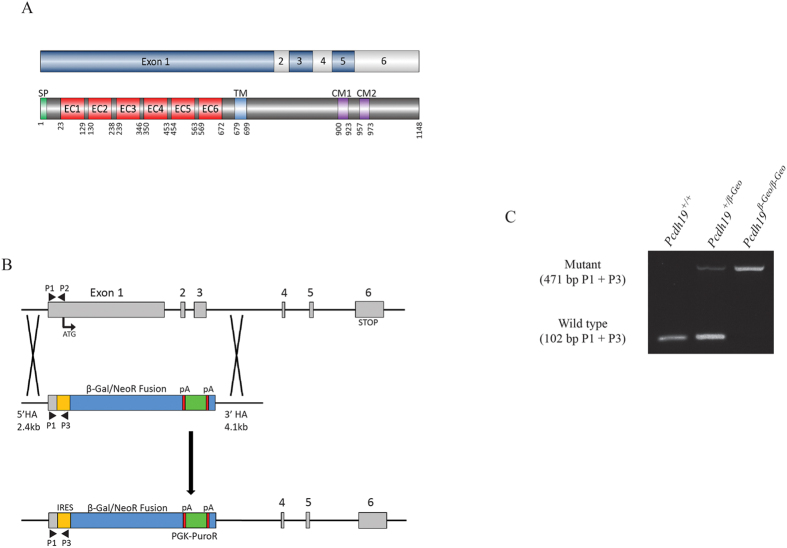
Generation of *Pcdh19* Null Mice. (**A**) *Pcdh19* contains 6 exons. Exon 1 encodes more than half of the protein including the entire extracellular domain and transmembrane domain. (**B**) *Pcdh19* null mice were generated by Taconic through targeted homologous recombination. Exon 1, 2 and 3 were removed and replaced with a *β-Galactosidase-Neomycin* fusion cassette. (**C**) PCR was used to confirm the presence of the β-Galactosidase/Neomycin fusion cassette with primers P1, P2 and P3 (black arrowheads in **B**). The *β-Galactosidase-Neomycin* fusion cassette was only present in *Pcdh19*^+/*β-Geo*^ and *Pcdh19*^Y/*β-Geo*^ mice.

The *Pcdh19* gene contains six exons, with exon 1 encoding the majority of the protein including the entire extracellular and transmembrane domains (Fig. 1A). To investigate the physiological role of *Pcdh19* and the molecular pathology of *PCDH19* GCE, we acquired a *Pcdh19* null mouse model in which exons 1–3 were replaced with a *β-geo* cassette (Fig. 1B). Removal of the first 3 exons leads to deletion of both the extracellular and transmembrane domains of *PCDH19*. The genetic disruption of *Pcdh19* gene was validated by PCR of genomic DNA (Fig. 1C) and ablation of *Pcdh19* expression confirmed by qPCR of 2 week old hippocampal cDNA (Supplementary Fig. 1).

**Figure 2:**
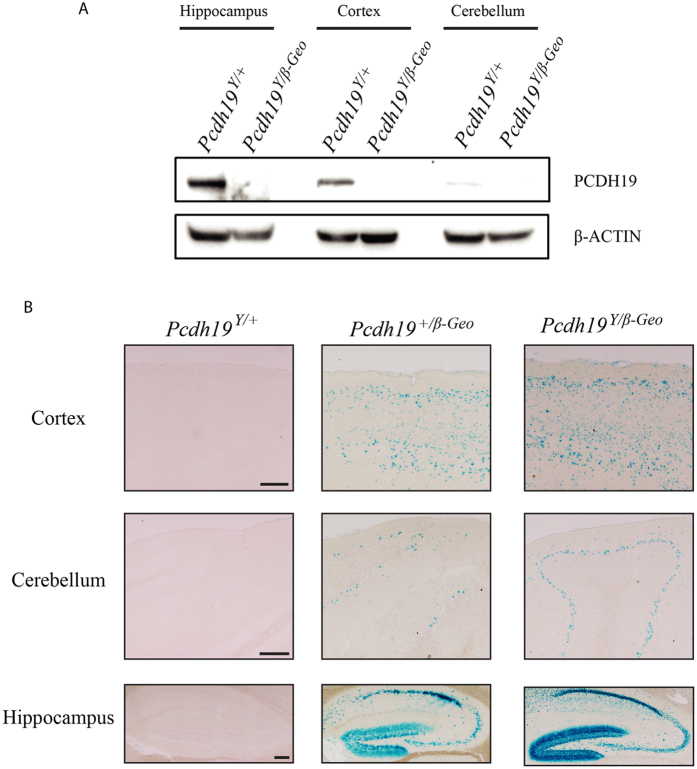
Validation of *Pcdh19* Null mice. (**A)** Protein extracts from hippocampus, cortex and cerebellum brain regions were immunoblotted with *PCDH19* antibody. *PCDH19* was detected in wild type extracts but was absent from *Pcdh19*^Y/*β-Geo*^ extracts. (**B**) Frozen sections from the same regions as A) were stained for β-Galactosidase activity using X-Gal. Staining was present in both *Pcdh19*^+/*β-Geo*^ and *Pcdh19*^*Y/β-Geo*^ brain sections but absent from wild type brain sections. Scale bar represents 250 μm.

Previous *in situ* hybridisation analysis has demonstrated *Pcdh19* expression in the hippocampus and cortex of postnatal mouse brains^5^. However, expression of endogenous *PCDH19* protein has not been shown. To investigate this issue, we performed western blot analysis using a commercially available *PCDH19* antibody. Consistent with mRNA expression data, robust expression was observed in the hippocampus and cortex (Fig. 2A).

Integration of the *β-geo* cassette into the *Pcdh19* locus potentially provides a reporter allele for null cells that would normally express *Pcdh19*. To confirm the activity of the *β-geo* cassette, X-Gal staining was performed on brain tissue from *Pcdh19*^*Y/β-Geo*^*, Pcdh19*^+/*β-Geo*^ and *Pcdh19*^+/Y^ mice*. Pcdh19*^*Y/β-Geo*^ brains exhibit positive staining in multiple brain regions including the hippocampus, cortex and cerebellum (Fig. 2B) which is consistent with western blot analysis (Fig. 2A) and previously published RNA expression data^5^. *Pcdh19*^+*/β-Geo*^ brains showed staining in the same brain regions but exhibited a mosaic staining pattern (Fig. 2B*). Pcdh19*^+/Y^ brains showed no staining. This confirms the *β-Geo* cassette is active and that *Pcdh19* undergoes random X-inactivation across multiple tissues similar to results in humans^19^.

**Figure 3:**
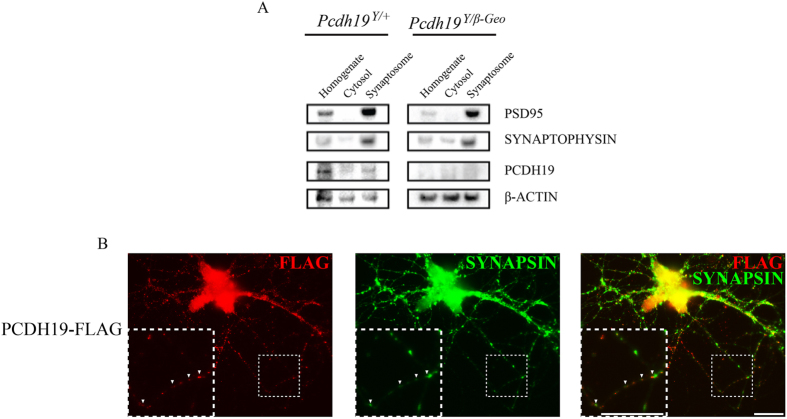
*PCDH19* is expressed in the synapses of hippocampal neurons. (**A)** Synaptosome fractionation of P14 mouse hippocampus was performed and immunoblotted for *PCDH19. PCDH19* protein was present in the synaptosome fraction which was also enriched for the synapse proteins PSD95 and SYNAPTOPHYSIN. *PCDH19* was absent from all *Pcdh19*^*Y/β-Geo*^ fractions. (**B**) Primary neurons were harvested from 18.5 dpc hippocampi and nucleofected with *PCDH19*-FLAG ORF. Immunostaning at DIV12 shows *PCDH19* is colocalised with SYNAPSIN positive synapses. Scale bar represents 20 μm.

Chemical fractionation of adult hippocampal samples revealed that *PCDH19* was present within the synaptosome, providing evidence for a role in synaptic function (Fig. 3A). Importantly, no signal was detected in *Pcdh19*^*Y/β-Geo*^ samples, further confirming *PCDH19* loss-of-function in this KO mouse model. Furthermore overexpression of FLAG tagged human *PCDH19* (*PCDH19*-FLAG) in primary hippocampal mouse neurons revealed *PCDH19* localisation with SYNAPSIN further supporting *PCDH19* expression in synapses (Fig. 3B).

### *Pcdh19* ^+*/β-Geo*^, *Pcdh19* ^*β-Geo/β-Geo*^ and *Pcdh19* ^*Y/β-Geo*^Mice have No Gross Phenotypic Abnormalities

Breeding of the *Pcdh19* mouse model generated *Pcdh19*^+*/β-Geo*^, *Pcdh19*^*β-Geo/β-Geo*^ and *Pcdh19*^*Y/β-Geo*^ mice at the expected frequencies (Supplementary Fig. 2). Adult mice with these genotypes had normal body size and did not exhibit any overt health issues (data not shown). To date, spontaneous seizures have not been detected in *Pcdh19*^+*/β-Geo*^, *Pcdh19*^*β-Geo/β-Geo*^ or *Pcdh19*^*Y/β-Geo*^ mice after weaning (ie. from 3 weeks of age).

**Figure 4:**
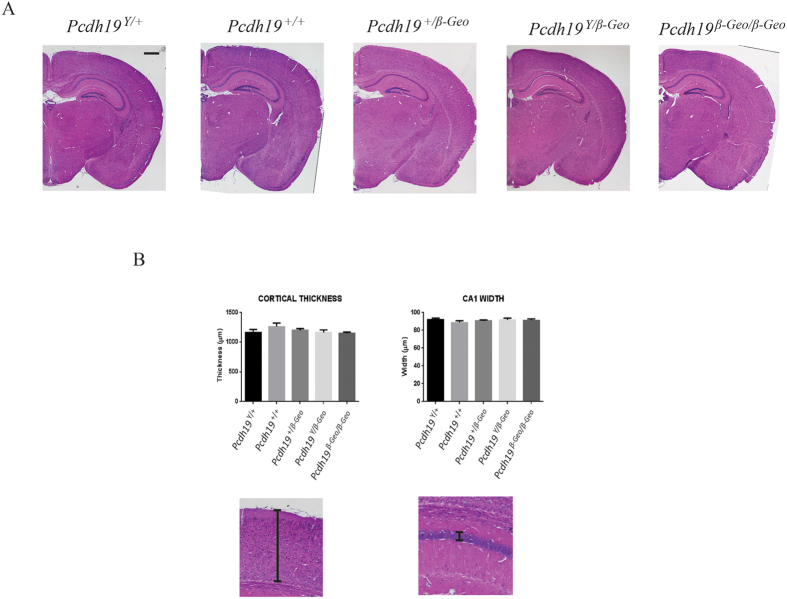
Histological and morphometric analysis of adult *Pcdh19* mutant mouse brains. (**A)** Haematoxylin and Eosin staining was performed on coronal P42 brain sections. Scale bar represents 1 mm. (**B)** Thickness measurement of the lateral parietal association cortex and hippocampus CA1 region in WT and *Pcdh19* mutant brains. Three animals were analysed for each genotype. Error bars = SEM.

Morpholino knock-down of *pcdh19* in zebrafish embryos results in profound CNS abnormalities. We therefore examined gross brain morphology in *Pcdh19* null mice. Histological analysis of *Pcdh19*^+*/β-Geo*^, *Pcdh19*^*β-Geo/β-Geo*^ and *Pcdh19*^*Y/β-Geo*^ brains showed no gross morphological abnormalities (Fig. 4A). As *Pcdh19* is highly expressed in the hippocampus and cortex, we performed morphometric analysis of these regions to determine if more subtle defects may be present. Measurements were made of the cortical thickness and CA1 region of the hippocampus with no significant difference observed in *Pcdh19*^+*/β-Geo*^, *Pcdh19*^*β-Geo/β-Geo*^ and *Pcdh19*^*Y/β-Geo*^ adult brains compared with *Pcdh19*^+/+^ and *Pcdh19*^Y/+^controls (Fig. 4B). We therefore conclude that mutation of *Pcdh19* in mice does not overtly affect brain structure.

### *Pcdh19* Null Cells are Located Normally in the Developing and Adult Brain

**Figure 5:**
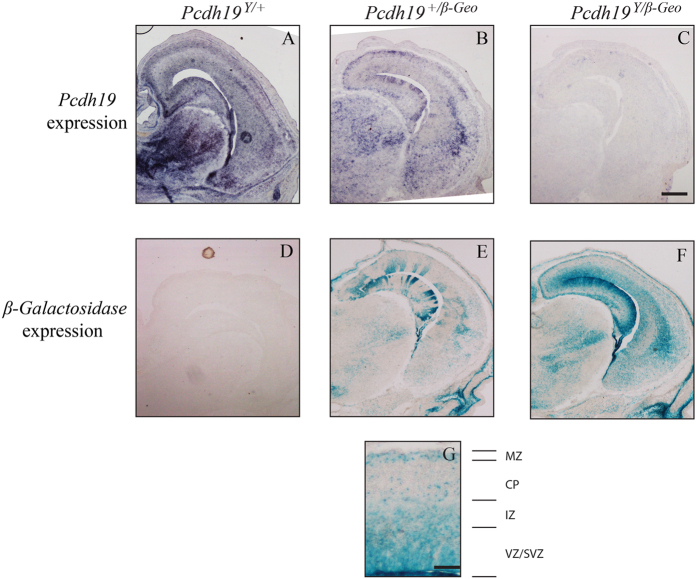
*Pcdh19* null cells are not ectopically located in the developing brain (15.5 dpc). **(A–C)** *Pcdh19* WT cells were detected by *in situ* hybridisation and were located in the cortex and hippocampus of *Pcdh19*^*Y*/+^(**A**) and *Pcdh19*^+/*β*-*Geo*^ (**B**) developing brains. *Pcdh19*^+/*β*-*Geo*^ brains exhibited less extensive staining consistent with X-inactivation. No specific signal was detected in *Pcdh19*^*Y/β*-*Geo*^ brains (**C**). (**D–F)** *Pcdh19* null cells were detected by X-Gal staining and were located in the cortex and hippocampus of *Pcdh19*^+/*β*-*Geo*^ (**E**) and *Pcdh19*^*Y/β*-*Geo*^ (**F**) developing brains. *Pcdh19*^+/*β*-*Geo*^ brains showed reduced staining consistent with X-inactivation. No signal was detected in *Pcdh19*^*Y/*+^brains (**D**). Scale bar represents 500 μm. Three animals were analysed for each genotype and age. (**G)** *Pcdh19* null cells are predominantly located in the ventricular zone (VZ)/subventricular zone (SVZ), the intermediate zone (IZ) and the marginal zone (MZ). Scale bar represents 100 μm.

Previous studies in zebrafish have shown that morpholino knockdown of *Pcdh19* results in severe disruption of brain morphogenesis due to an arrest of cell convergence in the anterior neural plate as well as abnormal cell migration during neurulation^11^,^13^, although this was not seen in *pcdh19* null zebrafish^12^. To investigate whether mutation of *Pcdh19* in mice affects neuronal migration during brain development we looked for ectopic localisation of *β-Geo* positive cells in developing (15.5 dpc) brains *of Pcdh19*^+/*β-Geo*^ and *Pcdh19*^*Y/β-Geo*^ mice using X-gal staining. As the *Pcdh19*-specific antibody used for western blot analysis was not compatible with immunohistochemistry (data not shown), wild type *Pcdh19*-expressing cells were identified by *in situ* hybridisation. At 15.5 dpc, robust *Pcdh19* expression was detected in the developing cortex and hippocampus of *Pcdh19*^*Y*/+^brains (Fig. 5A). Wild type *Pcdh19*-expressing cells were also present in the cortex and hippocampus of *Pcdh19*^+/*β-Geo*^ brains, although these were fewer in number consistent with X-inactivation (Fig. 5B). Importantly, analysis *of Pcdh19*^*Y/β-Geo*^ and *Pcdh19*^+/*β-Geo*^ embryonic brains revealed *β-Geo*-positive cells throughout the cortex and hippocampus in the same regions as wild type *Pcdh19* expressing cells (Fig. 5E,F). To confirm that the different methods used to detect *Pcdh19* expressing and mutant cells did not confound the analysis we performed *in situ* hybridisation of 15.5 dpc brains with a *β-Geo* probe (Supplementary Fig. 3). Cells expressing *β-Geo* were identified in the cortex and hippocampus as expected including the marginal zone, intermediate zone and ventricular/sub-ventricular zones (Fig. 5G). Taken together, these data suggest that mutant cells are not ectopically positioned in *Pcdh19*^+/*β-Geo*^ and *Pcdh19*^*Y/β-Geo*^embryonic brains.

**Figure 6:**
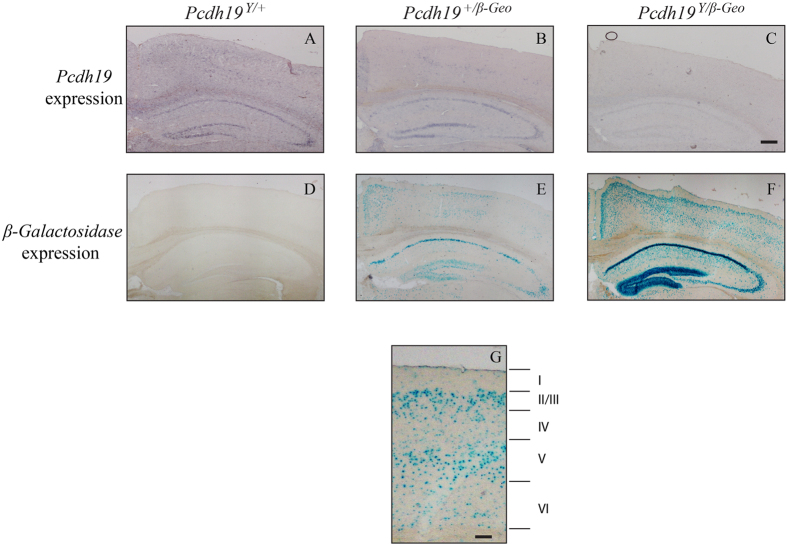
*Pcdh19* null cells are not ectopically located in the adult brain. **(A–C)** *Pcdh19* WT cells were detected by *in situ* hybridisation and were located in the cortex and hippocampus of *Pcdh19*^*Y*/+^(**A**) and *Pcdh19*^+/*β*-*Geo*^ (**B**) adult (P42) brains. *Pcdh19*^+/*β*-*Geo*^ brains exhibited less staining consistent with X-inactivation. No specific signal was detected in *Pcdh19*^*Y/β*-*Geo*^ brains (**C**). (**D–F)** *Pcdh19* null cells were detected by X-Gal staining and were located in the cortex and hippocampus of *Pcdh19*^+/*β*-*Geo*^ (**E**) and *Pcdh19*^*Y/β*-*Geo*^ (**F**) adult brains. *Pcdh19*^+/*β*-*Geo*^ brains showed less staining consistent with X-inactivation. No signal was detected in *Pcdh19*^*Y*/+^brains (**D**). Scale bar represents 500 μm. Three animals were analysed for each genotype and age. (**G)** *Pcdh19* null cells are predominantly expressed in layer II/III and V of the adult cortex. Scale bar represents 100 μm.

Given that *Pcdh19* expression is maintained after birth^5^, we next investigated whether *β-Geo* expressing cells were ectopically expressed in *Pcdh19*^+/*β-Geo*^ and *Pcdh19*^*Y/β-Geo*^brains at postnatal day 42 (P42). Consistent with our western blot analysis (Fig. 2A), robust expression of *Pcdh19* was detected in the hippocampus and cortex of *Pcdh19* WT brains (Fig. 6A). *Pcdh19* expression was strongest in the CA1 and dentate gyrus of the hippocampus and in layers 2/3 and 5 of the cortex. Mosaic expression was identified in the same regions of *Pcdh19*^+/*β-Geo*^ brains (Fig. 6B).

*β-Geo* staining of *Pcdh19*^*Y/β-Geo*^ brains with X-Gal revealed robust expression in the cortex and hippocampus that resembled the pattern of *Pcdh19* expression in *Pcdh19*^+/Y^ brains (Fig. 6). Mosaic expression of *β-Geo* was observed in these regions in *Pcdh19*^+/*β-Geo*^ brains (Fig. 6E). No evidence of ectopic *β-Geo*-positive cells was detected. Thus, it appears that the mutation of *Pcdh19* does not perturb positioning of neurons in postnatal brain development.

### The Migration Potential of *Pcdh19* Null Neurons is Increased *In Vitro*

**Figure 7:**
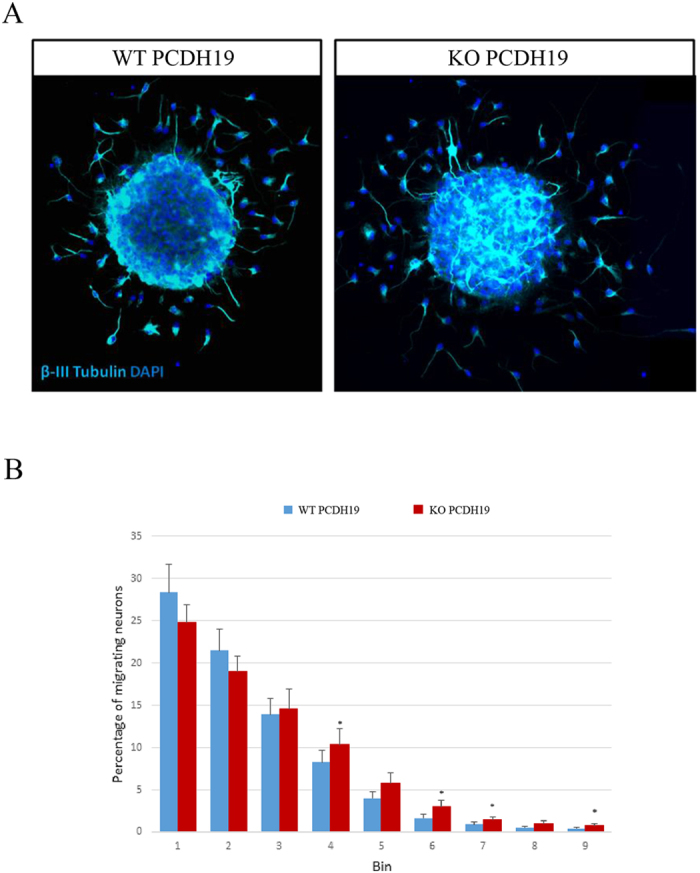
Loss of *Pcdh19* leads to an increase in neuronal migration. Neural progenitor cells (NPCs) were isolated from the telencephalic vesicles of *Pcdh19* WT or *Pcdh19* null E14.5 embryos and grown as non-adherent neurospheres. Neurospheres were plated on a Poly-L-Lysine substrate and cells were allowed to attach and migrate. (**A**) Neuronal migration was scored at 48 hrs by co-staining with a neuronal specific antibody β-III Tubulin (Cyan) and DAPI. (**B**) Neuronal migration was determined by drawing concentric circles around the contiguous boundary of the neurosphere and counting the number of neurons within each circle. Differences in the number of migrating neurons was determined by the percentage of neurons within each circle (bin). The percentage of neurons is greater in the more distal circles in the KO *Pcdh19* neurospheres compared to the WT *Pcdh19* neurospheres. Each data point represents 7 embryos collected from 3 litters with at least 20 neurospheres scored for each embryo. Error bars = SEM, *differs from WT; P < 0.05, Student’s T-test.

As subtle defects in neuronal migration can be difficult to detect *in vivo*, we next performed *in vitro* migrations assays using neurospheres generated from *Pcdh19* WT and *Pcdh19* null embryos. Neural progenitor cells were isolated from the developing cortex (14.5 dpc) and cultured as non-adherent neurospheres. To model neuronal migration, whole neurospheres were adhered to a poly-L-lysine substrate and the migration of neurons outward from the neurosphere boundary measured^20^. Increased neuronal migration in the *Pcdh19* null neurons was observed, as shown by an increase in the percentage of migrating neurons in the outer migration regions (bins) (Fig. 7A,B). However, this only reached significance in 4/9 of the bins. This data suggests that the mutant cells may have a subtle change in neuronal migration potential that does not translate into overt positional changes of neurons in the developing and adult mouse brain.

## Discussion

Mutations of protocadherin genes in humans have been implicated in several neurological diseases and in mice have revealed important functions in neuronal development including axon guidance, neural circuit formation and synaptogenesis. Here we performed validation and gross morphological characterization of *Pcdh19* null mice to determine the function of *Pcdh19 in vivo.*

Previous studies indicate that *Pcdh19* expression is temporally and spatially restricted within the embryonic and neonatal CNS^5^,^21^. Using *in situ* hybridization and *β-Geo* reporter activity, we have shown that *Pcdh19* expression is maintained in the adult brain. Expression was particularly robust in regions of the brain that are implicated in epilepsy including the hippocampus and in the cortex, where it was restricted to layers 2/3 and 5, although expression in other areas such as the cerebellum and the hypothalamus was also evident. We have attempted to further characterize endogenous *PCDH19* CNS protein expression by immunohistochemistry using several commercially available *PCDH19* antibodies, however none bind specifically to *PCDH19* as evident by the immunostaining patterns that were indistinguishable between *Pcdh19* WT and *Pcdh19* null brain sections (our unpublished data). The *β-Geo* reporter gene in our *Pcdh19* null mouse therefore provides a useful alternative method for identification of cells that normally express *Pcdh19*, especially given that loss of *Pcdh19* function does not overtly affect neuron localization *in vivo* (see below).

Expression *of Pcdh19* in the adult brain also raises the question of its subcellular localization. Using chemical fractionation and primary hippocampal neuron culture, we have shown that *PCDH19* is located within both the synaptosome fraction *in vivo* and synapses *in vitro* suggesting it has synaptic function. Consistent with these results, analysis of chick *PCDH19* in the optic tectum revealed substantial overlap with the synaptic marker Syntaxin^7^. Interestingly, *Pcdh17*, the closest related family member to *Pcdh19*, has recently been shown to play a role in synapse formation and synaptic vesicle assembly in murine corticobasal ganglia^9^. These data raise the possibility that *PCDH19* may perform a similar function *in vivo* and supports the hypothesis that abnormal synaptic communication between *PCDH19* WT and *PCDH19* mutant cells contributes to the pathobiology of *PCDH19* GCE^5^.

Previous reports have shown that morpholino knockdown or genetic mutation of *pcdh19* in zebrafish resulted in contrasting phenotypes^11^,^12^,^13^. In agreement with mutation of *pcdh19* in zebrafish by Cooper *et al.* we show here that *Pcdh19*^*Y/β-Geo*^, *Pcdh19*^*β-Geo/β-Geo*^ and *Pcdh19*^+/*β-Geo*^ mice are healthy, fertile and do not exhibit gross defects in brain morphology. These striking phenotypic differences in zebrafish may reflect lack of genetic compensation in response to acute morpholino *Pcdh19* knockdown in zebrafish which has been shown to account for similar differences in other studies^22^. In humans, hemizygous males do not exhibit intellectual disability or seizures indicating that brain development occurs relatively normally without *PCDH19*, perhaps due to functional compensation by other *PCDH19* family members^23^. The absence of morphological defects in *Pcdh19*^*Y/β-Geo*^and *Pcdh19*^*β-Geo/β-Geo*^mice is therefore not unexpected and suggests that *Pcdh19* functional redundancy also occurs in mice. In contrast, the majority of *PCDH19* heterozygous human females develop infantile seizures with variable cognitive defects, although there is no evidence of gross morphological defects affected individuals. It has been proposed that this phenotype results from “cellular interference” i.e. segregation of *PCDH19* expressing neurons and *PCDH19* mutant neurons in the developing brain development resulting in abnormal neural circuitry^5^,^23^. While there is little *in vivo* data to support this model, it is interesting to note that cortical dysplasia and clustering of dysplastic pyramidal neurons has been detected in a single female with *PCDH19* GCE^15^. Although we did not detect any evidence of cortical dysplasia or gross abnormalities in *Pcdh19*^+/*β-Geo*^ mice, we cannot exclude the possibility that subtle regionally-restricted defects may be present. Ryan *et al.* also detected dysplastic pyramidal cells ectopically positioned in the deep white layer, suggesting defective neuronal migration. To investigate this phenotype *in Pcdh19*^+/*β-Geo*^ mice, we determined the location *of Pcdh19* null cells using X-Gal staining in both developing and postnatal *Pcdh19*^+/*β-Geo*^ brains. However, no evidence of ectopic *Pcdh19* null cells was detected indicating that *PCDH19* is not critical for neuronal cell migration *in vivo*. In contrast, a modest but significant increase in migration was detected in *Pcdh19* null neurons generated from neurosphere explants. It is therefore possible that loss of *Pcdh19* function compromises the migration potential of some neurons but that this defect is subtle and not associated with overt morphological defects *in vivo*.

In summary, our characterization of *Pcdh19* null mice reveals that *Pcdh19* is widely expressed in the CNS but not required for brain development *per se*. However, given the significantly increased motility of *Pcdh19* null neurons *in vitro* and the identification of *Pcdh19* mutant phenotypes in other species, further detailed analysis of this mouse model may reveal subtle abnormalities in *Pcdh19* null brains.

## Methods

### Animals

*Pcdh19* null mice (TF2108) were purchased from Taconic Biosciences. Both male and female animals were used in this study. The day the vaginal plug was detected was designated as embryonic day 0.5 (0.5 dpc). The day of birth was designated postnatal day 0 (P0). Each group subjected to analysis contained at least three mice. All of the experiments were approved by The University of Adelaide Animal Ethics Committee and performed according to their ethical guidelines.

### Genotyping

Genotyping was performed by PCR amplification using three primers: one primer common to both *Pcdh19* WT and *Pcdh19* null alleles F-5′-AGTCCACTACCGACTCTGCTG -3′, one specific to the *Pcdh19* WT allele R-5′-CAAAGTTAGCCAGGCGGGAC-3′ and one specific to the *Pcdh19* null allele R-5′-AACTCACAACGTGGCACTGG-3′. PCR products were separated on a 1% agarose gel. *Pcdh19* WT and null allele products were 102 bp and 471 bp, respectively.

### Protein extraction, synaptosome fractionation and western blotting

Cortex, hippocampi and cerebellum for protein extraction were minced in extraction buffer (50 mM Tris, 150 mM NaCl, 1% NP40 (Roche) and 1% Triton X-100 (Sigma) and incubated at 4 degrees C for 30 minutes. Synaptosome fractions were isolated using Syn-PER synaptic protein extraction reagent (ThermoFisher Scientific) according to the manufacturer’s instructions. Lysates and fractions were separated on Invitrogen Bolt™ precast 4–12% polyacrylamide gels and transferred to PVDF membrane before being blotted. Antibodies and their corresponding dilutions were: mouse anti-*PCDH19* 1:100 (Abcam, ab57510) and rabbit anti-β-ACTIN 1:1000 (Cell Signalling Technology, #4967), mouse anti-PSD95 1:2000 (ThermoScientific, 7E3-1138) and rabbit anti-SYNAPTOPHYSIN 1:5000 (Abcam, ab32127). Membranes were blocked in 5% BSA^+^5% Skim milk in Tris-buffered saline^+^0.1% Tween 20 (TBST) and antibodies were incubated with the membrane in 5% BSA in TBST. Membranes were developed using Bio-Rad Clarity Western ECL substrate and imaged using a Bio-Rad ChemiDoc.

### Primary Hippocampal Neuron Culture, Nucleofection and Immunohistochemistry

18.5 dpc hippocampi were dissected from wild type embryos and neurons dissociated with 0.5% Trypsin. 1 × 10^5^ neurons were nucleofected with 1 μg *PCDH19*-FLAG using the Neon Transfection System (Invitrogen) and seeded onto poly-D-lysine coated coverslips. Primary neurons were maintained in Neurobasal medium^+^B27 supplement (Life Technologies). Primary neurons were cultured for 12 days then fixed in 4% PFA for 15-30 minutes. For immunofluorescence, cells were block permeablised with 0.1% Tween20 (PBST) and 10% foetal calf serum for 1 hour at room temperature. Primary neurons were then incubated overnight with anti-FLAG (1:1000, Sigma Aldrich) and anti-SYNAPSIN (1:500, Millipore) at 4˚C and then with secondary antibody (donkey anti-mouse Cy3 and donkey anti-rabbit Cy5; JacksonImmunoResearch). Images were acquired on a Nikon Eclipse Ti microscope.

### X-gal staining

15.5 dpc embryonic heads and dissected P42 brains were fixed in 4% paraformaldehyde at 4 °C, cryoprotected in 30% sucrose and frozen in OCT embedding medium. Embryos were fixed in 4% paraformaldehyde in PBS. Whole embryos or cryosections were incubated in staining solution: 19 mM Sodium dihydrogen phosphate, 81 mM Disodium hydrogen phosphate, 2 mM MgCl_2_, 5 mM EGTA, 0.01% Sodium deoxycholate, 0.02% NP-40, 5 mM Potassium ferricyanide, 5 mM Potassium ferrocyanide, and 1 mg/ml X-gal substrate, at 37 °C until blue staining was sufficient. Images were acquired on a Nikon Eclipse Ti microscope, compiled and minimally processed (adjusted for color and light/dark) using Adobe Photoshop CS.

### Histological analysis

Embryos for histological examination were fixed in 4% PFA, embedded in paraffin, cut at 5μm and stained with Mayer’s haematoxylin and Eosin using standard approaches. Images were acquired on a Nikon Eclipse Ti Microscope and measurements taken using Nikon Imaging Software (NIS Elements). Three animals were analysed for each genotype with cortical measurements being made in the visual cortex and hippocampal measurements in the CA1 region. A total of ten measurements for each region was recorded for each individual animal and averaged. The mean was then determined from the average of the three animals in each group.

### In situ hybridisation

15.5 dpc embryonic heads and dissected P42 brains were fixed in 4% paraformaldehyde at 4 °C, cryoprotected in 30% sucrose and frozen in OCT embedding medium. *In situ* hybridization of 16-μM sections was performed as described previously^24^. The *Pcdh19* probe was a digoxigenin-labeled antisense RNA probe prepared as described^21^. The *LacZ* probe sequence (500 bp) was generated by PCR using the following primers F-5′-ATGTGCGGCGAGTTGCGTGA-3′ R-5′-CGCTCATCGCCGGAGCCAGC-3′. At least three independent samples of each group were analyzed and representative sections are shown. No signal was detected using a sense control probe. Images were acquired on a Nikon Eclipse Ti microscope, compiled and minimally processed (adjusted for color and light/dark) using Adobe Photoshop CS.

### Neurosphere Migration Assay

NPCs were isolated from the 14.5 dpc embryonic mouse cortex (telencephalic vesicles) and grown as non-adherent neurospheres in the presence of 20 ng/mL bFGF and EGF as previously described^25^. Passage 4 neurospheres were cultured for 5 days and seeded onto a poly-L-lysine substrate at a density of 100 neurospheres/9.5 cm^2^ with the removal of growth factors. Cells were allowed to migrate for 48 hrs before being fixed in 4% paraformaldehyde (PFA) in phosphate buffered saline (PBS) for 15 minutes at room temperature. For immunofluorescence analysis, cells were block permeablised in 0.2% tween20 (PBST) and 5% horse serum for 1 hour at room temperature. To identify neurons, cells were stained overnight with β-III tubulin primary antibody (1:300, Sigma Aldrich) in PBST with 0.5% horse serum at 4 °C and then with secondary antibody, donkey anti-mouse AlexaFluor647 (1:700, Invitrogen) for 1 hr at room temperature. Cells were counterstained with 300 nM 4′,6-diamidino-2-phenylindole (DAPI) to identify single cells and mounted with Slow-fade mounting media (Invitrogen) for microscopy. Fluorescence was viewed using the Zeiss AxioImager M2 fluorescent microscope (Carl Zeiss, Germany). Images of isolated single neurospheres and migrating cells were captured at 20× magnification using an Axiocam Mrm camera and Vs4.9.1.0 software (Axiovision, Carl Zeiss). Neurospheres were reconstructed using the Image J Stitching plugin^26^ and migration analysis preformed using the ImageJ2 software^27^. Briefly, the neurosphere boundary was identified and the radius of the neurosphere determined. The concentric circles plugin was used to draw 10 concentric circles (ie. migration zones) around the boundary of the sphere, with an outer radius calculated by the radius of the neurosphere^+^600 pixels (307 μM). Using the cell counter plugin the number of migrating neurons within each concentric circle was determined by counting co-stained β-III tubulin and DAPI cells. The migration distance was then calculated as a percentage of total migrating cells within each circle (bin). Each data point represents 7 embryos collected from 3 litters with at least 20 neurospheres scored for each embryo. Analysis conducted by Student’s two-tailed, unpaired t test, assuming equal variance.

### Real time PCR

RNA was extracted from P14 hippocampi by using an RNeasy mini column (Qiagen) according to the manufacturer’s instructions. Reverse transcription was performed on 1μg of RNA using a High Capacity RNA to cDNA synthesis kit (Life Technologies). Real time PCR was performed on a Step One Plus thermocycler (Life Technologies) using Fast Sybr green master mix (Life Technologies) according to manufacturer’s instructions on a two-step program. Primers and product sizes were as follows; *Pcdh19* (130 bp) F-5′ -TGGCAATCAAATGCAAGCGT-3′ R-5′ ACCGAGATGCAATGCAGACA-3′, β-Galactosidase (85 bp) F-5′ TACGATGCGCCCATCTACAC-3′ R5′ AACAACCCGTCGGATTCTCC-3′ β-actin (89 bp) F-5′-CTGCCTGACGGCCAGG-3′ R-5′-GATTCCATACCCAAGAAGGAAGG-3′.

## Additional Information

**How to cite this article**: Pederick, D. T. *et al.* *Pcdh19* Loss-of-Function Increases Neuronal Migration *In Vitro* but is Dispensable for Brain Development in Mice. *Sci. Rep.* **6**, 26765; doi: 10.1038/srep26765 (2016).

## Supplementary Material

Supplementary Information
