# Supplementary material for: Pcdh19 Loss-of-Function Increases Neuronal Migration In Vitro but is Dispensable for Brain Development in Mice
Source: Sci Rep. 2016 May 31;6:26765. doi: 10.1038/srep26765 (PMC4886214; doi:10.1038/srep26765)
Supplement: Supplementary Information [file srep26765-s1.pdf]

Supplementary Information

Pcdh19 Loss-of-Function Increases Neuronal Migration *In Vitro* but is Dispensable for Brain Development in Mice

Daniel T. Pederick, Claire C. Homan, Emily J. Jaehne, Sandra G. Piltz, Bryan P. Haines, Bernhard T. Baune, Lachlan A. Jolly, James N. Hughes, Jozef Gecz and Paul Q. Thomas

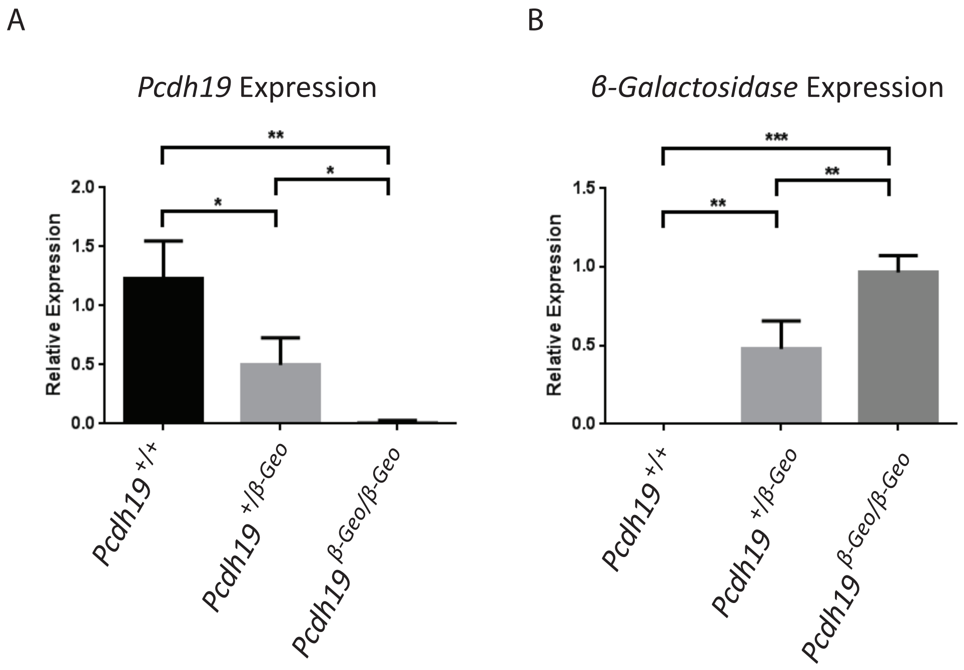

Supplementary Figure 1 Validation of Pcdh19 KO allele by quantitative PCR

A) Pcdh19 WT cDNA could be detected in P14 hippocampal cDNA extracted form Pcdh19+/+and Pcdh19+/β-Geo brains. A significant decrease in Pcdh19 WT cDNA was observed in Pcdh19+/β-Geo brains. Pcdh19 β-Geo/β-Geo samples had no detectable Pcdh19 WT cDNA. Statistical significance was analysed by one way ANOVA. Pcdh19+/+ vs Pcdh19+/β-Geo $p<0.05$, Pcdh19+/+ vs Pcdh19 β-Geo/β-Geo $p<0.01$ and Pcdh19+/β-Geo vs Pcdh19 β-Geo/β-Geo $p<0.05$. Performed with three biological samples of each genotype and in technical triplicates. Normalised to ActB.

B) β-Galactosidase cDNA could be detected in P14 hippocampal cDNA extracted form Pcdh19+/β-Geo and Pcdh19 β-Geo/β-Geo brains. A significant decrease in β-Galactosidase cDNA was observed in Pcdh19+/β-Geo brains. Pcdh19+/+ samples had no detectable β-Galactosidase cDNA. Statistical significance was analysed by one way ANOVA. Pcdh19+/+ vs Pcdh19+/β-Geo $p<0.01$, Pcdh19+/+ vs Pcdh19 β-Geo/β-Geo $p<0.001$ and Pcdh19+/β-Geo vs Pcdh19 β-Geo/β-Geo $p<0.01$. Performed with three biological samples of each genotype and in technical triplicates. Normalised to ActB.

A

		+/Y (M)	
		**+**	**Y**
**+/- (F)**	**+**	**+/+ 24.3%** (26)	**+/Y 22.4%** (24)
	**-**	**+/- 29.0%** (31)	**-/Y 24.3%** (26)

TOTAL 100%
(107)

B

		-/Y (M)	
		**-**	**Y**
**+/- (F)**	**+**	**+/- 22.0%** (17)	**+/Y 24.7%** (19)
	**-**	**-/- 28.6%** (22)	**-/Y 24.7%** (19)

TOTAL 100%
(77)

-

Supplementary Figure 2 Breeding of Pcdh19 null Mice produced all genotypes at the expected frequencies
A) A random sample of offspring generated from a Pcdh19+/β-Geo crossed with a Pcdh19Y/+ generated all genotypes at expected frequencies. Statistical significance was analysed by a Chi Square test p=0.8013.
B) A random sample of offspring generated from a Pcdh19+/β-Geo female crossed with a Pcdh19Y/β-Geo generated all genotypes at expected frequencies. Statistical significance was analysed by a Chi Square test p= 0.8820.

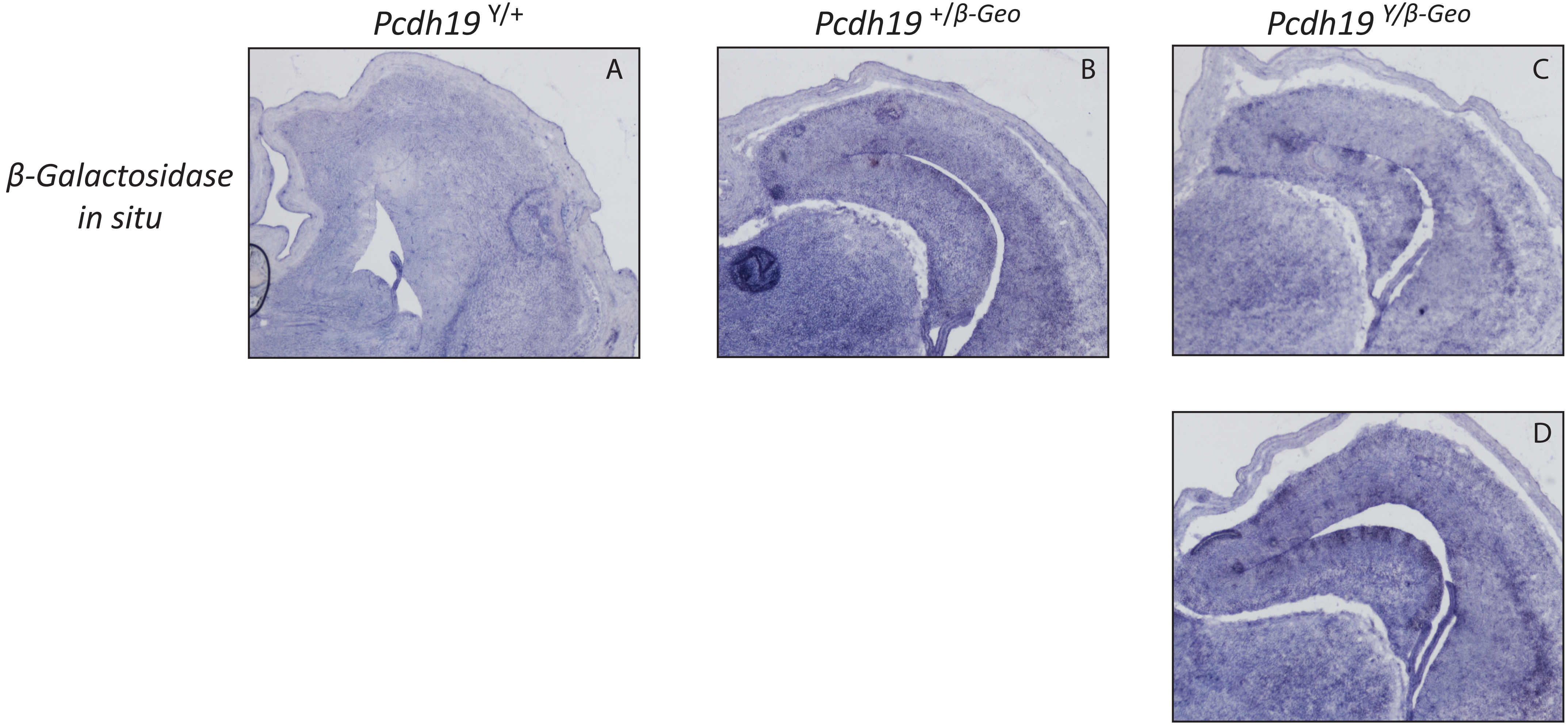

Supplementary Figure 3 Pcdh19 null cells were identified using in situ hybrisation and expressed in the developing cortex and hippocampus (E15.5)
A) Pcdh19 null cells were not present in Pcdh19Y/+ brains.
B) Pcdh19 null cells were present in the developing cortex and hippocampus of Pcdh19Y/β-Geo brains.
C-D) Pcdh19 null cells were the present in the developing cortex and hippocampus of Pcdh19+/β-Geo brains. Pcdh19+/β-Geo brains exhibited less staining consistent with X-inactivation.
